# CMP-sialic acid synthetase in *Drosophila* requires N-glycosylation of a noncanonical site

**DOI:** 10.1016/j.jbc.2025.108483

**Published:** 2025-04-07

**Authors:** Boris Novikov, Devon J. Boland, Ilya Mertsalov, Hilary Scott, Saniya Dauletbayeva, Pedro Monagas-Valentin, Vladislav Panin

**Affiliations:** 1 Department of Biochemistry and Biophysics, AgriLife Research, Texas A&M University, College Station, Texas, USA; 2 Texas A&M Institute of Genome Sciences & Society, Texas A&M University, College Station, Texas, USA; 3 Koltzov Institute of Developmental Biology, Russian Academy of Sciences, Moscow, Russia; 4 Department of Molecular Biology and Genetics, al-Farabi Kazakh National University, Almaty, Republic of Kazakhstan

**Keywords:** CMP-sialic acid synthetase, *Drosophila*, glycosylation, noncanonical N-glycosylation, sialylation

## Abstract

Sialylation plays important roles in animals, affecting numerous molecular and cell interactions. In *Drosophila*, sialylation regulates neural transmission and mediates communication between neurons and glia. *Drosophila* CMP-sialic acid synthetase (CSAS), a key enzyme of the sialylation pathway, is localized to the Golgi and modified by N-glycosylation, suggesting that this modification can affect CSAS function. Here, we tested this hypothesis using *in vitro* and *in vivo* approaches. We found that CSAS proteins from divergent *Drosophila* species have two conserved N-glycosylation sites, including the rarely glycosylated noncanonical N-X-C sequon. We investigated CSAS glycosylation by generating CSAS “glycomutants” lacking glycosylation sites and analyzing them *in vivo* in transgenic rescue assays. The removal of noncanonical glycosylation significantly decreased CSAS activity, while the canonical site mutation did not affect CSAS function. Although all glycomutants were similarly localized to the Golgi, the non-canonical glycosylation, unlike the canonical one, affected CSAS stability *in vivo* and *in vitro*. Our results suggested that CSAS functions as a dimer, which was also supported by protein structure predictions that produced a dimer recapitulating the crystal structures of mammalian and bacterial counterparts, highlighting the evolutionary conservation of the CSAS structure–function relationship. This conclusion was supported by the rescue of *CSAS* mutants using the human ortholog. The noncanonical CSAS glycosylation was discussed in terms of a potential mechanism of temperature-dependent regulation of sialylation in poikilotherms that modulates neural activity in heat shock conditions. Taken together, we uncovered an important regulation of sialylation in *Drosophila*, highlighting a novel interplay between glycosylation pathways in neural regulation.

Sialylation is a prominent type of glycosylation that affects numerous molecular and cell interactions in a wide range of organisms, including bacteria and animals (1, 2, 3). Sialylation plays critical roles in the mammalian nervous system. Essential functions of neural sialylation in humans are evident from prominent phenotypes of defects in the sialylation pathway, such as mutations in sialyltransferases (ST3GAL5 and ST3GAL3), CMP-Sia transporter (SLC35A1), CMP-Sia synthetase (CMAS), and Sia synthase (NANS) that are associated with severe neurological defects, including epilepsy, ataxia, and intellectual disability (4, 5, 6, 7, 8). However, the pathogenic mechanisms of these disorders are complex and remain poorly understood.

Among animals, *Drosophila* represents the earliest evolutionary branch of species with experimentally confirmed activity of the sialylation pathway, which makes *Drosophila* a useful model to reveal conserved mechanisms of sialylation (9, 10). Several key genes involved in sialylation have been characterized in fruit flies, including *Drosophila* sialyltransferase (DSiaT), CMP-sialic acid synthetase (CSAS, also known as CMAS or CSS in vertebrates), and sialic acid (Sia) synthase (NANS) (10, 11, 12, 13, 14). In *Drosophila*, the sialylation pathway is specifically upregulated in the nervous system where it mediates interactions between neurons and glia and plays important roles in regulating neural transmission, responses to oxidative stress, and promoting the function of voltage-gated sodium channels (15, 16, 17).

Although *Drosophila* CSAS shows prominent structural and functional homology to vertebrate counterparts, it has different subcellular localization from the vertebrate orthologues. *Drosophila* CSAS localizes in the secretory compartment, predominantly inside the Golgi, while vertebrate CSAS orthologs are localized in the nucleus (13, 16, 18), which represents a unique example of a drastic change during animal evolution of the subcellular localization of an enzyme with a conserved biological function. Unlike vertebrate orthologues, due to its localization inside the secretory compartment, *Drosophila* CSAS is modified with N-linked glycosylation, suggesting that glycosylation can play a role in CSAS regulation (19). In this study, we shed light on CSAS glycosylation and its effect on CSAS function using *in vivo* and *in vitro* approaches. We identified glycosylation sites and characterized the function of individual glycans by site-directed mutagenesis of N-glycan attachment sites. CSAS proteoforms with removed glycosylation sites (“glycomutants”) were analyzed *in vivo* using rescue assays, which revealed different requirements of distinct glycans for CSAS function. Our results shed light on the molecular structure of the CSAS, suggesting that CSAS functions as a homodimer, which was also supported by *in silico* modeling of the CSAS structure. The CSAS molecular model showed remarkable similarity to the crystal structures previously obtained for mammalian and bacterial homologs, suggesting that *Drosophila* CSAS employs enzymatic mechanisms similar to these evolutionarily distant counterparts.

## Results

### Two N-glycosylation consensus sites are present in many CSAS orthologs in *Drosophila* species

**Figure 1:**
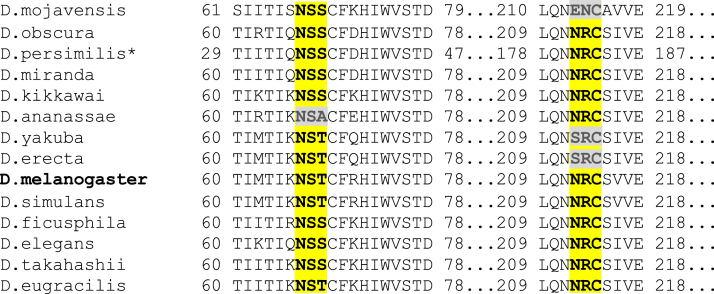
**Evolutionary conservation of predicted N-glycosylation sites in CSAS sequences from different *Drosophila* species.** The canonical (N-X-S/T) and noncanonical (N-X-C) sites are highlighted. Multiple sequence alignment was performed using Clustal Omega (60). CSAS sequences were obtained from the OrthoDB catalog of orthologs (59). ∗The sequence of *Drosophila persimilis* CSAS is missing a part of the N terminus due to incomplete sequencing information. CSAS, CMP-sialic acid synthetase.

Our previous experiments revealed that *Drosophila* CSAS is a glycoprotein localized to the secretory pathway compartments (16, 19). To explore the function of CSAS glycosylation, we first analyzed the CSAS amino acid sequence for the presence of possible sites for N-linked glycosylation. We identified two potential sites within the predicted luminal part of the enzyme. One of them, located closer to the N terminus, has a usual consensus sequence for N-linked glycosylation, N-X-T, while another site, located further downstream, closer to the C terminus, is N-X-C, a rarely used noncanonical site of glycosylation (20). Interestingly, these sites are well-conserved in CSAS sequences of different *Drosophila* species estimated to have diverged about 30 million years ago (21), suggesting that CSAS glycosylation plays important functional roles in fruit flies (Fig. 1). The CSAS glycosylation sites are not conserved in vertebrates (*e.g.*, Fig. S1), which are consistent with their localization outside of the secretory compartment.

### Both predicted N-glycosylation sites are utilized in *Drosophila* CSAS but with different efficiency

**Figure 2:**
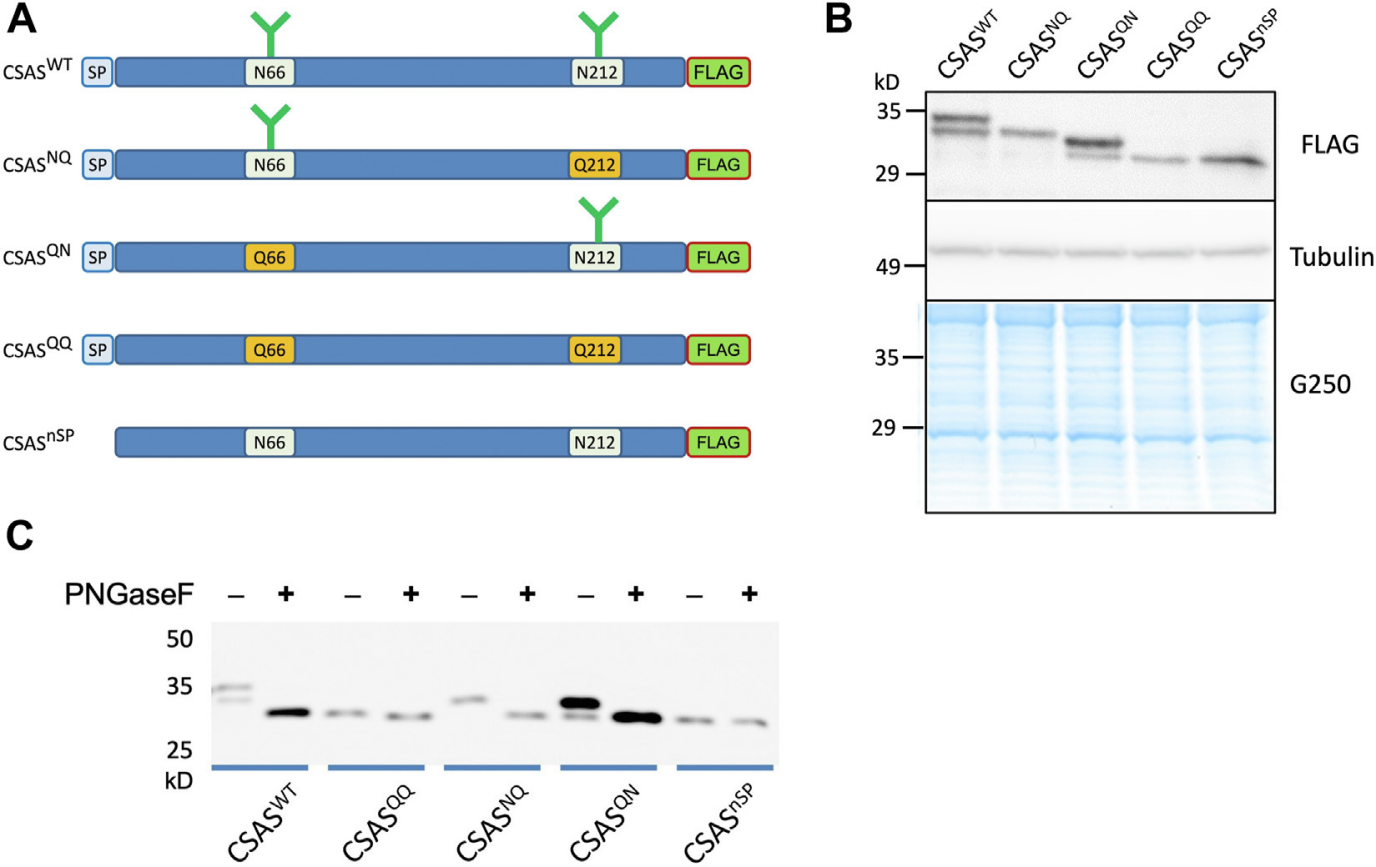
**Generation and *in vivo* expression of mutagenized CSAS constructs.***A*, schematic of CSAS constructs. The constructs are depicted in N- to C-terminus orientation. SP, signal peptide. N66 and N212 indicate the canonical and noncanonical site of N-glycosylation, respectively. FLAG, a 3xFLAG tag. Predicted N-glycans are depicted by *green* antennal shapes. *B*, expression of CSAS constructs in flies. The expression was detected by FLAG antibody using Western blot (*top* panel). Tubulin antibody western and gel protein staining with Coomassie G250 (*low* panel) were used as loading controls (*middle* panel). The constructs were expressed using *Act5C-Gal4* driver in WT flies (see Supplementary Information for a complete description of genotypes). Young adult flies (0–2 day-old) were used for the analyses of transgenic expression. *C*, treatment of CSAS constructs with PNGaseF that removes N-glycans. CSAS, CMP-sialic acid synthetase.

To examine the glycosylation of the potential sites, we used site-directed mutagenesis to create CSAS expression constructs encoding N- > Q substitutions at the predicted glycosylation sites. These mutations should eliminate possible glycosylation while having minimal effect on the structure of the CSAS protein. In addition to single-site mutants, designated as CSAS^NQ^ and CSAS^QN^, we generated a double mutant with both predicted sites mutated (CSAS^QQ^), as well as a CSAS construct with truncated signal peptide (CSAS^nSP^) (Figs. 2*A*, S1). The CSAS^nSP^ mutant is expected to be localized outside of the secretory compartments and thus not carrying N-glycans. These mutant coding DNA (cDNA) constructs were tagged with a 3xFLAG-encoding sequence at the 3′ end and cloned into the *pUASTattB* vector for UAS-Gal4 system-mediated *in vivo* expression (22). The mutant constructs, along with 3xFLAG-tagged wild-type *CSAS* construct (*CSAS*^*WT*^), were introduced as transgenes using PhiC31-mediated integration in the same attP2 landing site on the third chromosome (23, 24). The insertion into the same genomic location ensured that the expression of these constructs is not affected by the position effect, and thus the constructs can be quantitatively compared in transgenic expression experiments (25, 26). First, we expressed these constructs using a ubiquitous driver (*Act5C-Gal4*), and analyzed their expression by Western blots. The comparison of constructs’ gel mobility revealed that mutation in either predicted glycosylation site resulted in a decrease of molecular mass consistent with the elimination of an N-glycan, while the double mutant showed an additional decrease in molecular mass, suggesting that both glycosylation sites are indeed modified by N-glycans (Fig. 2*B*). These blots also revealed that the efficiency of glycosylation is different for distinct sites. While the canonical site was always modified, about 40% of the CSAS protein was not glycosylated at the noncanonical site, based on the intensity of additional bands for CSAS^WT^ and CSAS^QN^ revealed by Western blots (Fig. 2*B*). The CSAS^nSP^ mutant is expected to be devoid of N-glycosylation because the lack of signal peptide should prevent it from entering the endoplasmic reticulum. Indeed, this mutant did not show a size difference on the gel from QQ double mutant, which also suggested that CSAS does not have any additional glycosylation besides the glycans attached to the two predicted N-glycosylation sites. We also performed the treatment of CSAS glycoforms with peptide-N-Glycosidase F (PNGase F), a glycosidase that specifically removes N-linked glycans by cleaving between the innermost GlcNAc and asparagine residues of attachment sites. The removal of glycans resulted in a shift of the WT, NQ, and QN glycoforms on the gel, down to the size of the nonglycosylated QQ glycomutant (Fig. 2*C*). As expected, no effect of PNGase F treatment was detected on QQ and nSP. These conclusions were consistent with the results of endoglycosidase H (Endo H) treatments (Fig. S2). Endo H cuts N-glycans between the two GlcNAc residues in the chitobiose core but cannot work on complex structures (27). Thus, the sensitivity of CSAS glycoforms to Endo H also revealed that CSAS lacks processed N-glycans, which is consistent with the fact that processed glycan structures are exceedingly rare in *Drosophila* (12, 28). Together, these results confirmed that CSAS is modified by N-linked glycans at two glycosylation sites, and that the sites are modified with different efficiency, resulting in two glycoforms detected on the gel by Western blot for WT and QN.

### Glycosylation at different sites has a distinct effect on CSAS activity *in vivo*

**Figure 3:**
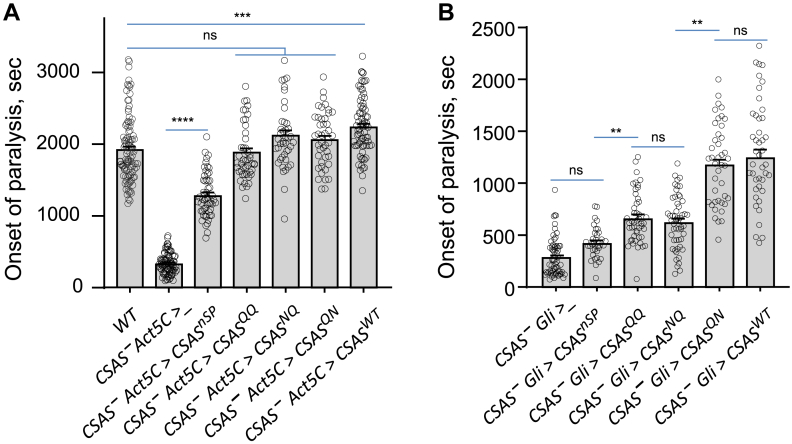
**Rescue of the TS paralysis phenotype of *CSAS* loss-of-function mutants by transgenic expression of CSAS constructs.***A*, CSAS constructs were expressed in *CSAS* mutants using *Act5C-Gal4* driver. *CSAS*^*–*^*Act5C>_*, the control *CSAS* mutant genotype including only the driver but no constructs (“driver-only” control). *B*, CSAS constructs were expressed in *CSAS* mutants at a moderate level using *Gli-Gal4* driver and the TARGET system (all genotypes included *Tub-Gal80ts*). See Supplementary material for a complete description of the genotypes. ns, no significant difference (*p* > 0.05); ∗∗, *p* < 0.01; ∗∗∗, *p* < 0.001; ∗∗∗∗, *p* < 0.0001. CSAS, CMP-sialic acid synthetase; TS, temperature-sensitive; WT, wild type control.

We next tested the activity of CSAS mutant constructs *in vivo* using rescue approach. The constructs were ectopically expressed in *CSAS* mutants by UAS-Gal4 system, and their activities were analyzed by the ability to rescue the temperature-sensitive (TS) paralysis phenotype, a characteristic feature of *Drosophila* sialylation mutants (15, 16, 17). When we induced the expression of constructs using *Act-Gal4*, a strong ubiquitous driver, we found that all three glycomutants, CSAS^NQ^, CSAS^QN^, and CSAS^QQ^, could fully rescue TS paralysis, while the expression of CSAS^nSP^ resulted in a partial rescue (Fig. 3*A*). The ectopic expression of CSAS^WT^ using *Act-Gal4* produced flies that were more resistant to heat shock than WT flies (Fig. 3*A*). This result is consistent with previous data indicating that CSAS activity is limited *in vivo*, representing a bottleneck of the sialylation pathway, and that CSAS overexpression can render flies more resistant to environmental stresses than WT flies (17). In order to analyze the activity of CSAS constructs at a low level of expression that more closely reflects the endogenous expression, we repeated rescue experiments using conditions with a decreased level of transgenic expression. To this end, we used *Gli-Gal4*, a weaker driver that was previously shown to fully rescue *CSAS* mutants in transgenic expression experiments (17), combined with the expression of Gal80ts, a TS mutant form of the transcription repressor Gal80 which was designed to precisely regulate the UAS-Gal4 expression. Gal80ts was shown to have a gradually decreasing activity when temperature increases between 18 °C (full activity) and 32 °C (complete inactivation), thus affording to control the level of UAS-Gal4-mediated expression by temperature (the TARGET system (29)). We assayed the rescue activity of CSAS glycomutants using the *Gli-Gal4–Gal80ts* combination at 28 °C, a temperature causing a partial suppression of *Gli-Gal4*-mediated expression by Gal80ts. This condition avoided the rescue “saturation” due to overexpression, providing a more sensitive assay to compare the activities of different CSAS constructs. At these nonsaturating conditions, only the CSAS^QN^ mutant showed activity indistinguishable from WT CSAS. The activities of CSAS^NQ^ and CSAS^QQ^ mutants were substantially lower, while being not significantly different from each other (Fig. 3*B*). The CSAS^nSP^ mutant showed no significant activity in rescuing *CSAS* mutants, suggesting that localization to the secretory pathway compartment is important for CSAS function (Fig. 3*B*). Taken together, these results uncovered differences between the activities of CSAS glycosylation mutants, which revealed that mutation of the noncanonical site impaired the activity of CSAS, while the glycosylation of the canonical site showed no significant effect on CSAS activity.

### Mutations of glycosylation sites do not significantly affect the subcellular localization of CSAS glycomutants

**Figure 4:**
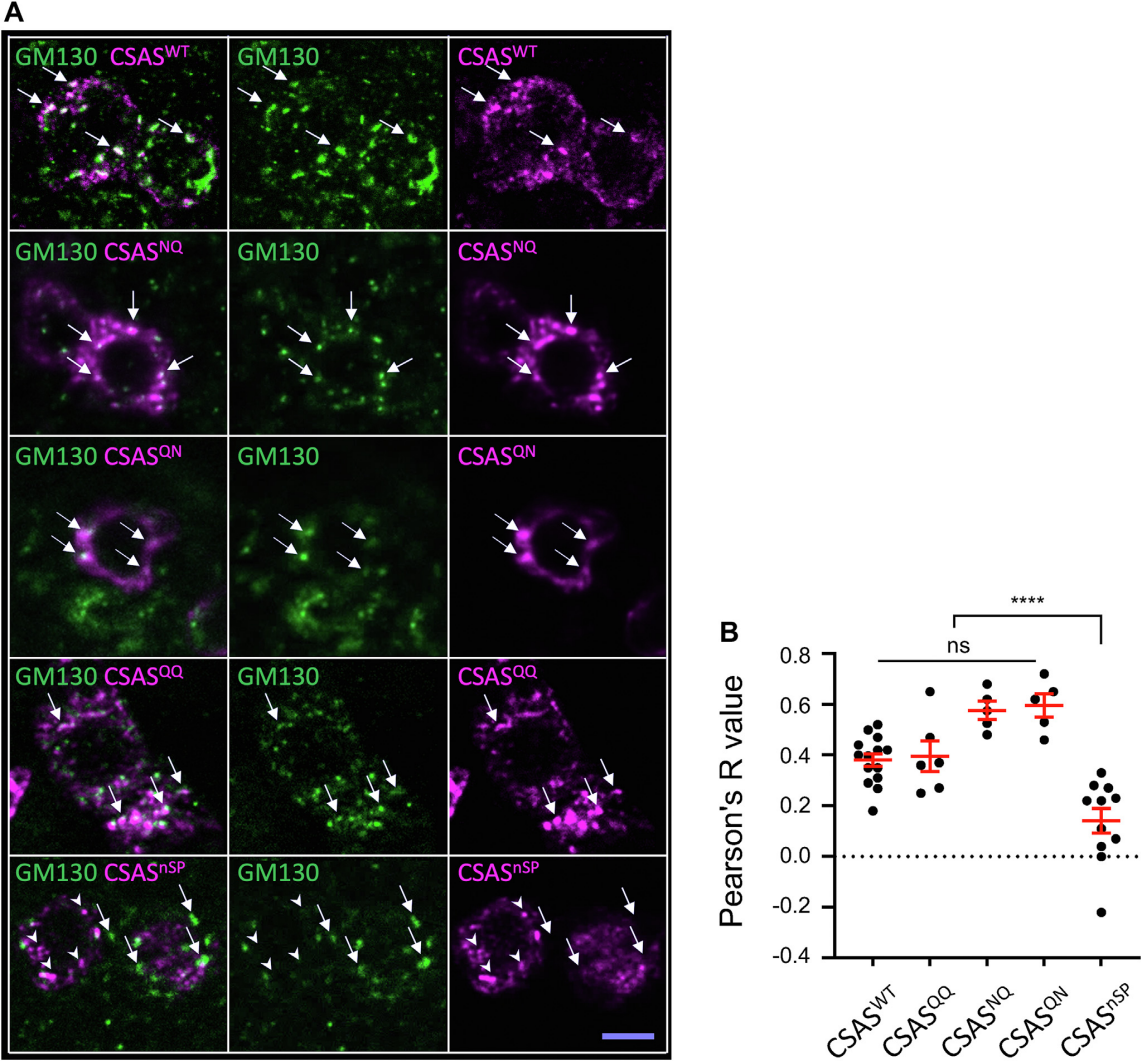
**Subcellular localization of CSAS constructs.***A*, representative fluorescent images of optical sections through *Drosophila* larval brain immunostained using GM130 and FLAG antibodies. In the *top* four rows of panels, *arrows* indicate examples of colocalization between GM130 and FLAG epitopes. In the last row that shows CSAS^nSP^, *arrows* and *arrowheads* indicate examples of nonoverlapping localization of GM130 and CSAS^nSP^, respectively. Scale bar is 5 μm. *B*, colocalization of CSAS constructs with GM130 was quantified by Pearson’s R value. All contracts showed significant colocalization with GM130 except for CSAS^nSP^ that had minimal overlap with GM130. ns, no significant difference (*p* > 0.05), ∗∗∗∗, *p* < 0.0001. CSAS, CMP-sialic acid synthetase.

To investigate possible mechanisms underlying the activity differences among CSAS glycoforms, we examined whether glycans affect CSAS localization within the secretory pathway compartment. Previous studies have demonstrated that CSAS predominantly resides in the Golgi while also being present throughout the secretory compartment (16, 30). Using immunofluorescent staining and confocal microscopy, we analyzed the colocalization of CSAS glycomutants with the Golgi marker GM130 (31) (Fig. 4*A*). The subcellular localization of all glycomutants exhibited similar prominent overlap with GM130, with no significant differences in Pearson's correlation coefficients (Fig. 4*B*). Their staining patterns extended beyond GM130, a marker for cis-Golgi, consistent with previous findings that CSAS localizes throughout the secretory compartment. These results suggest that glycosylation does not significantly affect CSAS subcellular localization, indicating that the activity differences between CSAS glycomutants cannot be attributed to altered subcellular distribution. In contrast, CSAS^nSP^ showed minimal colocalization with GM130 (Pearson's correlation coefficients, 0.11 ± 0.05), supporting the predicted localization outside the secretory compartment.

### Evolutionary conservation of CSAS function between *Drosophila* and mammals

**Figure 5:**
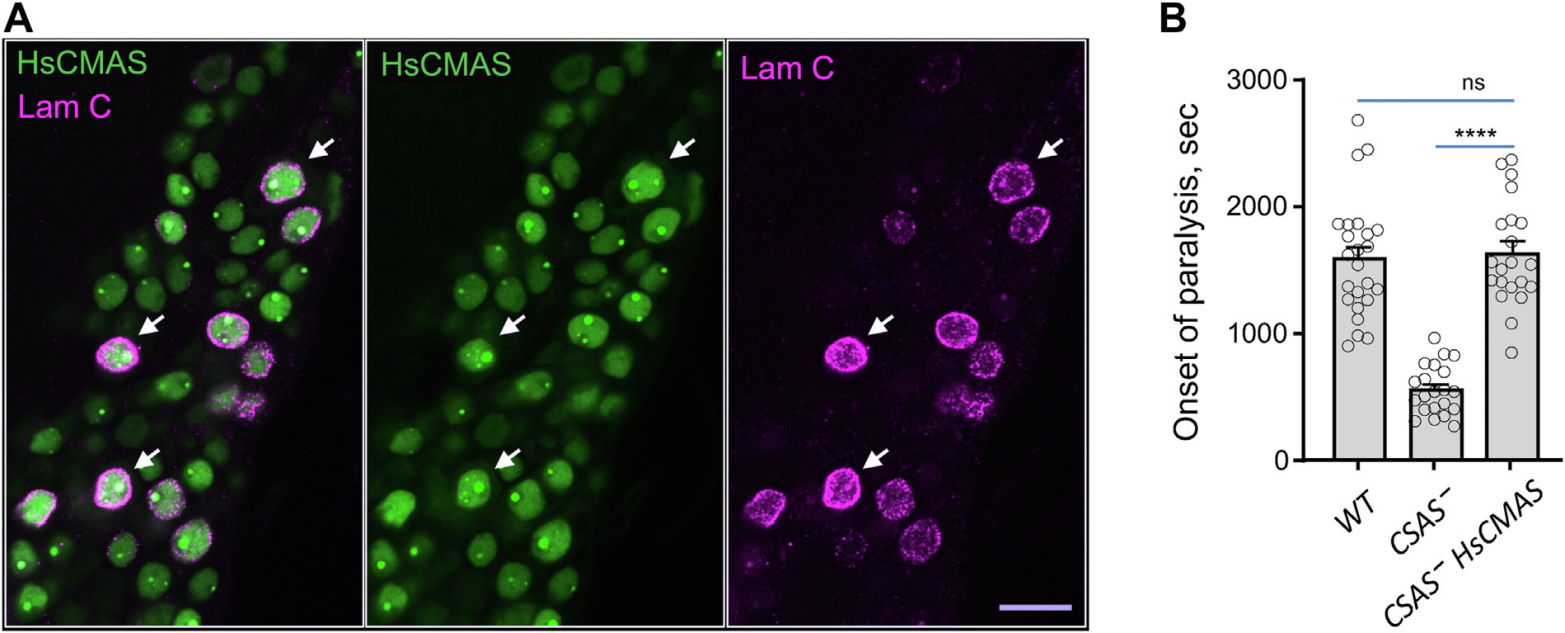
***In vivo* expression of human CMAS can rescue *CSAS* mutants.***A*, human CMAS is localized largely to the nucleus when expressed in *Drosophila* brain cells. *Green*, HsCMAS immunofluorescent staining; *magenta*, labeling nuclei with Lamin C, a marker for the nuclear envelope (70). *Arrows* indicate examples of nuclear envelope staining (*red*) surrounding HsCMAS localized inside the nucleus (*green*, *arrowheads*). Scale bar is 10 μm. *B*, HsCMAS can fully rescue TS paralysis phenotype of CSAS mutants. WT, wild-type control flies, *CSAS*^–^, homozygous mutant flies, *CSAS*^*–*^*HsCMAS, CSAS* mutants with transgenic expression of human CMAS. ns, no significant difference (*p* > 0.05); ∗∗∗∗, *p* < 0.0001. CMAS, CMP-Sia synthetase; CSAS, CMP-sialic acid synthetase; TS, temperature-sensitive.

Interestingly, although CSAS^nSP^, the mutant form localized outside of the secretory compartment, showed no *in vivo* activity when expressed at a lower level, it could provide partial rescue when overexpressed (Fig. 3*A*). This suggests that the mechanism of transferring CMP-Sia across the membrane from the cytoplasm into the Golgi still exists in *Drosophila*, although being not very efficient. To shed more light on this scenario and test the evolutionary conservation of CSAS function between fruit flies and mammals, we transgenically expressed the human ortholog of *Drosophila* CSAS, HsCMAS, in *CSAS* mutants. Mammalian orthologs of CSAS, also termed CMAS proteins, have nuclear localization signals and localize to the nucleus in mammalian cells (32). We found that HsCMAS was similarly localized to the nucleus in *Drosophila* cells (Fig. 5*A*). Despite this distinct subcellular localization compared to the *Drosophila* counterpart, the transgenic HsCMAS fully rescued *CSAS* mutants (Fig. 5*B*), which revealed the evolutionary conservation of CMP-Sia synthetase function between fruit flies and humans. HsCMAS provided more efficient rescue than CSAS^nSP^ (Figs. 3*A* and 5*B*), which is probably explained by a significantly higher enzymatic activity of the human CMAS compared to its *Drosophila* orthologue (19). These results also confirmed that CMP-Sia can be transported into the Golgi from outside of the secretory compartment, providing evidence of CMP-Sia transporter activity in *Drosophila*.

### The stability of CSAS is decreased without glycosylation of the noncanonical site

**Figure 6:**
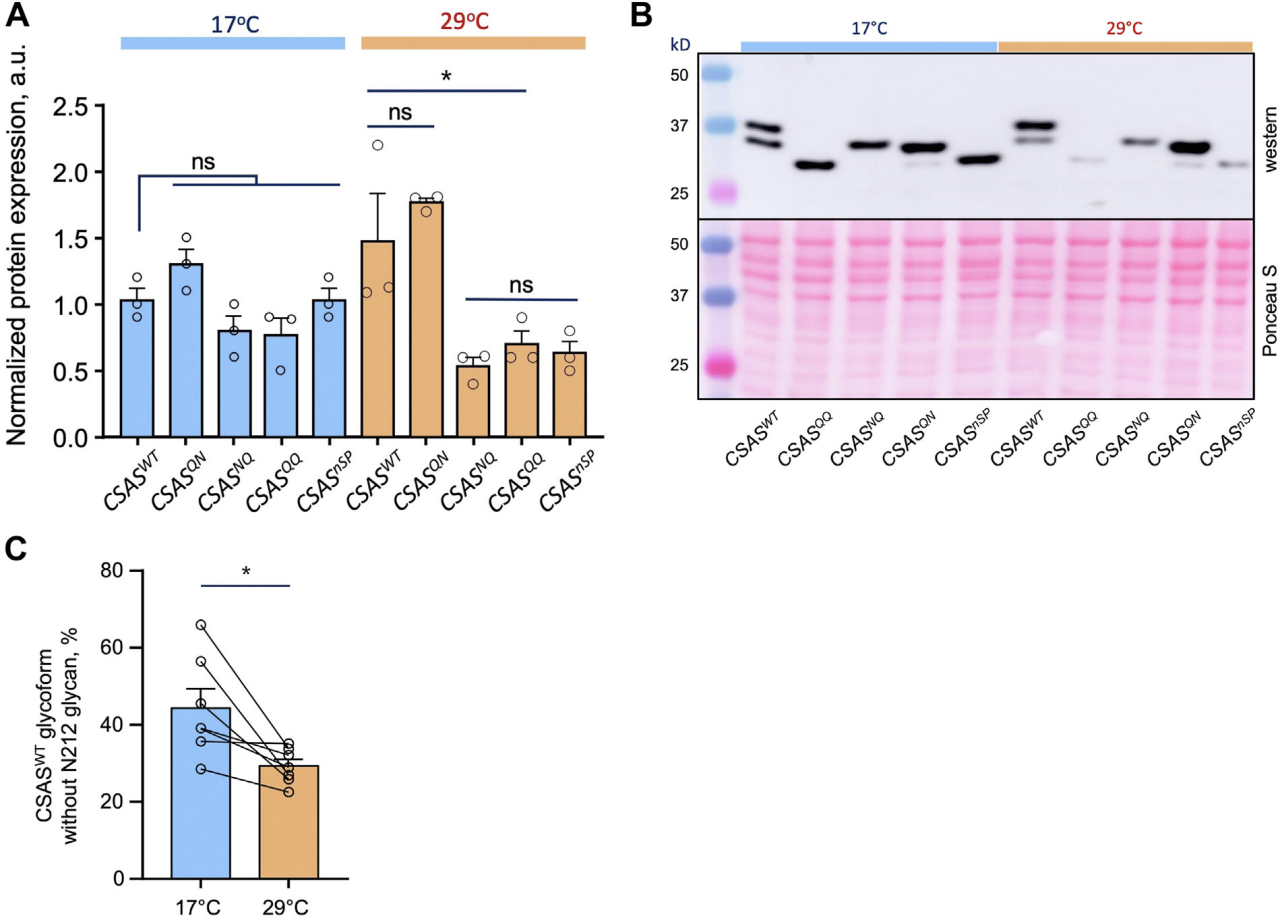
**Expression level of CSAS constructs at different temperatures.***A*, quantitative comparison of different CSAS constructs expressed at 17 °C and 29 °C by Western blots. The Western blot signal was normalized to the total protein amount estimated by Ponceau S staining. a.u., arbitrary units. *B*, a representative example of a Western blot and Ponceau S staining. *C*, the relative amount of the glycoform of CSAS^WT^ without glycosylation of the noncanonical site decreases at elevated temperature, as revealed by Western blot analysis. *Lines* connect data obtained in the same experiment/biological repeat. *A*–*C*, results were obtained using at least three biological replicates, each including several technical repeats. ∗*p* < 0.05; ns, no significant difference (*p* > 0.05). CSAS, CMP-sialic acid synthetase.

The difference in activity of different CSAS glycoforms can potentially result from differences in their stability. To further test this hypothesis, we analyzed the level of expression of CSAS glycomutants at different temperatures. Considering that all transgenic constructs were inserted into the same landing site, multiply outcrossed to the same genetic background (*w- Canton S*), induced by the same *Gal4* driver, and analyzed in flies of the same age, the differences in their level of protein expression are expected to reflect the *in vivo* stability of these proteins. We quantified the expression of CSAS glycomutants induced by a ubiquitous driver using Western blots at two different temperatures, 17 °C and 29 °C, to account for a possible effect of temperature on protein stability. While no significant differences in protein expression level were found at 17 °C, the relative level of WT and QN was significantly higher than that of other constructs at 29 °C (Fig. 6, *A* and *B*). Furthermore, the proportion of the glycoform without glycosylation of the noncanonical site was significantly decreased at elevated temperature (Fig. 6*C*). These results indicated that the stability of CSAS is compromised without glycosylation of the noncanonical site. The rescue activity of CSAS glycomutants correlated with their protein expression level (Figs. 3*B* and 6*A*), suggesting that a decreased protein stability underlies the diminished activity of the glycomutants without the noncanonical glycosylation site.

**Figure 7:**
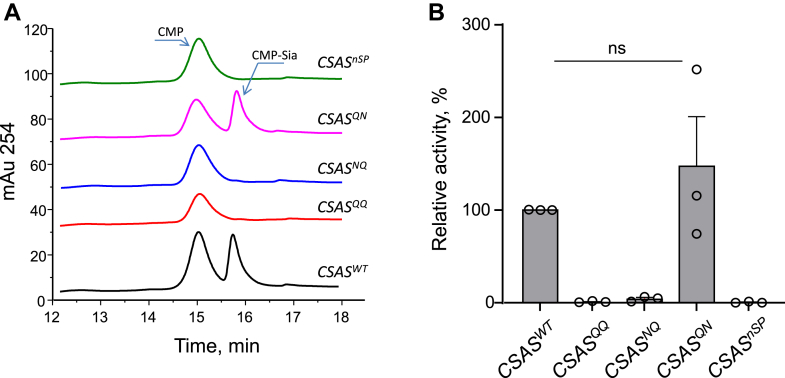
***In vitro* analysis of enzymatic activity of purified CSAS constructs.***A*, example traces of UV detector signal of chromatographic separation of CMP-sialic acid synthetase reactions. CMP and CMP-Sia peaks are indicated (CMP is produced by nonspecific hydrolysis of CTP in the reaction mixture). *B*, quantification of CSAS constructs’ relative activities. The relative activities were calculated based the ratio of CMP-Sia produced in the assay to the relative amount of the CSAS variant added to the assay (estimated by Western blot), normalized to the relative activity of CSAS^WT^. No *in vitro* activity was detected for purified CSAS^QQ^, CSAS^NQ^, and CSAS^nSP^ constructs. CSAS, CMP-sialic acid synthetase.

We also examined the *in vitro* activity of CSAS glycomutants. To this end, we purified CSAS proteins from flies with transgenic expression of constructs using FLAG affinity beads and assayed CMP-Sia synthetase activity using *in vitro* assays (19). CSAS^WT^ and CSAS^QN^ proteins showed similar activities, while no activity was detected for CSAS^NQ^, CSAS^QQ^, and CSAS^nSP^ (Fig. 7). These results suggested that the stability of the CSAS functional form is compromised without glycosylation of the noncanonical site and that these CSAS glycoforms lost their activity during purification, possibly due to unfolding and/or aggregation. This conclusion is consistent with *in vivo* experiments suggesting that glycosylation of the noncanonical site promotes CSAS stability (Fig. 6).

### CSAS protein forms dimeric complexes

**Figure 8:**
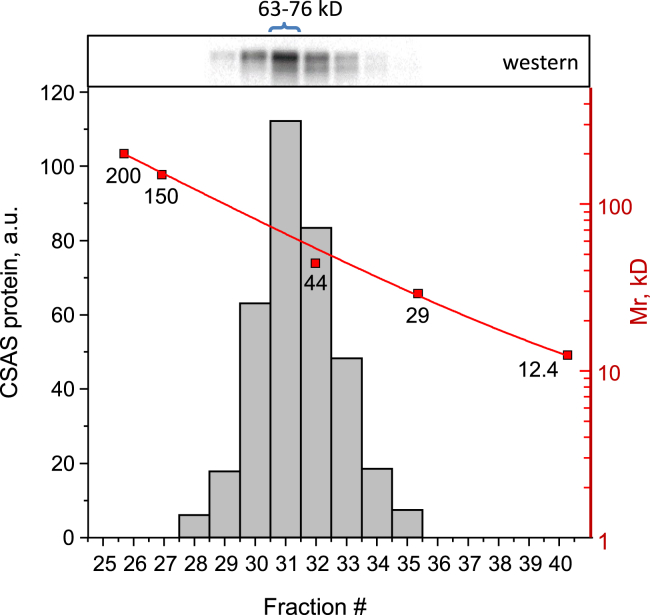
**Analysis of CSAS molecular complexes by size-exclusion chromatography.** Protein lysates of *Drosophila* with transgenic expression of CSAS-FLAG were separated by size-exclusion chromatography and the molecular weight of CSAS complexes was estimated by Western blot analysis of collected fractions. The histogram shows the amount of CSAS present in different fractions obtained by separation on a calibrated column. The calibration curve is overlaid on the histogram with molecular mass standards indicated (shown in *red*). The *top* panel shows CSAS-FLAG in corresponding fractions analyzed by FLAG Western blot of collected fractions (Fig. S3 shows a full image of the membrane). The estimated molecular mass corresponding to the peak fraction of the histogram is indicated. CSAS, CMP-sialic acid synthetase.

**Figure 9:**
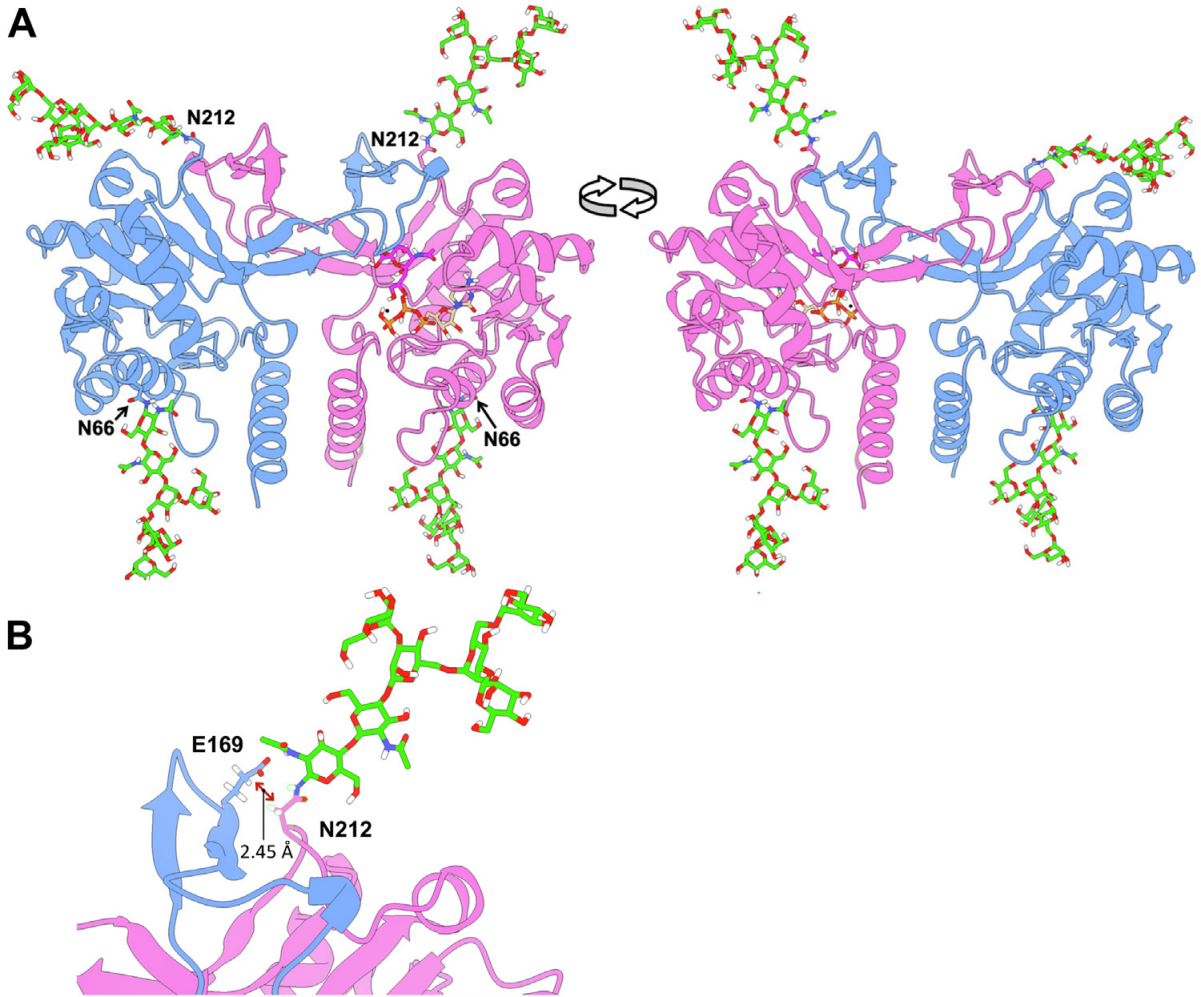
**Molecular modeling of CSAS dimers by AlphaFold2 and metal/substrate docking.***A*, two views of the 3D CSAS dimer model obtained by ∼180^o^ rotation. Individual monomers are shown in *blue* and *pink*. Mg^2+^ ion (*black sphere*) and substrates, sialic acid (*purple*) and CTP (*orange* and *pale green*), are shown in the active site of one monomer (*pink*). Man_5_GlcNAc_2_ oligomannose N-glycans (M5N2) are shown in *spring green*. The glycans were grafted at N66 and N212 residues on both monomers using CHARMM program (glycans are flexible and may acquire various conformations). The atoms shown in *white*, *blue*, *red*, and *orange* are H, N, O, and P. *B*, a zoomed-in view of the N212 glycan attachment site on one monomer (*pink*) and a fragment of the dimerization region of another monomer (*blue*). The estimated distance between N212 and E169 residues of different monomers is indicated. CSAS, CMP-sialic acid synthetase.

N-terminal catalytic domains of mammalian CMP-Sia synthetases form functional dimers stabilized by intertwined dimerization regions, which is thought to be important for their *in vivo* activity (8, 33). *Drosophila* CSAS shows significant homology to the N-terminal domains of mammalian counterparts (13, 19), suggesting that CSAS can also form dimers. To test this possibility, we performed size-exclusion chromatography of cell lysates obtained from flies with the transgenic expression of CSAS-FLAG. Western blot analysis of fractions including different molecular sizes revealed that the CSAS protein exists as complexes with a molecular weight in the range of 63 to 76 kD, which is consistent with the dimer configuration (Fig. 8). To further explore the hypothesis of CSAS dimerization, we carried out three-dimensional protein structure prediction using AlphaFold2 (34). The dimerization of CSAS was predicted with high confidence, revealing dimers with an intertwined structure (Fig. 9). To reveal the catalytic site, we carried out molecular docking with Mg^2+^ as a metal cofactor, Neu5Ac and CTP as substrates, and CMP-Sia synthetase structure from *Vibrio cholerae* as a reference structure (35). The resulting 3D model of *Drosophila* CSAS closely recapitulated the crystal structures of counterpart enzymes from *Neisseria meningitidis* and mouse (33, 36). The molecular model indicated that the utilization of both glycosylation sites is compatible with the dimer stricture as the sites are localized on the surface of the dimer and the attachment of N-linked glycan is not expected to interfere with the predicted molecular structure. We modeled the presence of oligomannose glycans, the most prevalent N-linked structures in *Drosophila* (12, 28), attached to the predicted glycosylation sites of the dimer using CHARMM (37). The glycan chains are predicted to be located on the CSAS dimer far from the catalytic sites and not be able to obstruct interactions with substrates. However, the noncanonical glycosylation site is located in proximity of the dimerization region (Fig. 9), suggesting that its glycosylation may affect the CSAS dimer’s stability, which is consistent with our analysis of CSAS glycoforms.

## Discussion

CMP-sialic acid synthetase is thought to be the bottleneck of the sialylation pathway in animal cells (38), which suggests that this enzyme can be involved in the regulation of sialylation. Indeed, *in vivo* experiments in *Drosophila* indicated that CSAS KO mutations are dominant as CSAS heterozygotes show decreased resistance to heat, while CSAS overexpression in otherwise WT flies increases their tolerance to heat and oxidative stress (17). The regulation of CSAS function within the cell is complex and not well-understood. Our previous studies indicated that *Drosophila* CSAS is modified with N-linked glycans, suggesting that they may be important for the function of this enzyme. Here, we explored this possibility by characterizing the glycosylation sites and the function of glycans that they bear. We found that CSAS is modified at two N-glycosylation sites that are highly conserved among *Drosophila* species diverged in evolution as much as humans and lizards (39), which suggests the functional significance of these modifications. Intriguingly, one of these sites is the rarely used noncanonical N-X-C site which is modified in animal cells with a frequency as low as 1.3% (40). So far, the glycosylation of the noncanonical site was confirmed for a small number of proteins using glycoproteomic approaches, and its functional importance was analyzed on few glycoproteins in cell culture (41, 42, 43, 44, 45), but this type of glycosylation was not previously investigated *in vivo*. Here, we examined and compared the function of the canonical and noncanonical CSAS glycosylation sites *in vivo* using rescue assays that analyzed the ability of different CSAS glycoforms to restore the function of the sialylation pathway in *CSAS* mutants.

Interestingly, the removal of the sites by mutagenesis did not affect the ability of transgenically expressed CSAS to rescue the phenotype of *CSAS* mutants when the constructs were overexpressed at a high level, which indicated that N-linked glycosylation is not essential for CSAS catalytic activity. However, when the CSAS glycomutants were expressed at a moderate level, more closely reflecting the endogenous CSAS expression, their ability to restore the function of the sialylation pathway was significantly different. The lack of the noncanonical glycosylation site significantly decreased the CSAS *in vivo* activity, while the removal of the canonical site had no significant effect on CSAS activity in the context of the WT protein or the glycomutant without noncanonical site (Fig. 3*B*). Although we did not detect the effect of the canonical site mutation in our experiments, it should be noted that the rescue assay that we used to compare the CSAS glycoforms may not be sensitive or specific enough to discern more subtle effects caused by the mutation. As suggested by its evolutionary conservation, the canonical site glycosylation is potentially also important for some CSAS functions which, however, are not essential for maintaining the heat shock tolerance of flies.

The conservation of the noncanonical glycosylation site in CSAS proteins is remarkable among *Drosophila* species and extends to diverse arthropods beyond the *Drosophila* genus, appearing in species as distant as butterflies and crabs (Fig. S4). CSAS in arthropods is predicted to localize to the secretory pathway compartment, suggesting that the function of the noncanonical glycosylation may be conserved across a wide range of invertebrate species. However, besides *Drosophila*, the secretory compartment localization of CSAS has been experimentally confirmed only for two other insect species, a mosquito and a beetle—though whether their CSAS proteins have noncanonical N-glycosylation remains unknown (30). Interestingly, the noncanonical site is not universally conserved among *Drosophila* and other arthropod species (Fig. 1*A*), suggesting that its function can be mediated through alternative mechanisms, such as potential glycosylation at nonconserved sites or through specific protein structural features. A similar scenario was described for CD2, a conserved T-lymphocyte adhesion molecule that requires N-glycosylation for functional stability in humans. While this glycosylation is conserved in mice, it is absent in rats, where the stability is achieved *via* specific features of the polypeptide chain instead (46). It will be interesting to investigate whether analogous mechanisms operate in the CSAS protein family.

N-linked glycosylation can influence protein functions by affecting subcellular trafficking and localization (20, 47). The effect of noncanonical site glycosylation on human B4GALNT2 glycosyltransferase was studied in cell culture, which indicated that this glycosylation could affect activity, stability, and subcellular localization of the enzyme (48). In our study, we did not detect a significant effect of glycosylation on CSAS localization, based on colocalization analysis with GM130, a Golgi marker with which all glycoforms showed similar significant overlap. Although we cannot exclude a possibility that glycosylation can affect CSAS localization in a more subtle way which was not discerned by our experiments, together with the analyses of *in vivo* expression and activity of purified proteins, our results strongly suggest that the noncanonical glycosylation is mainly important for the stability of CSAS, which is consistent with previous studies showing that N-linked glycosylation can promote folding and enhance stability of proteins (49, 50, 51). This conclusion is supported by several lines of evidence. First, the mutation of the noncanonical site significantly decreased the steady-state expression level of the CSAS protein at elevated temperature, which correlated with the diminished *in vivo* activity of CSAS constructs lacking the noncanonical glycosylation (Figs. 3*B* and 6). Secondly, purification of the noncanonical site glycomutants resulted in enzymatically inactive proteins. At the same time, purified glycomutant without the canonical site showed similar activity to that of WT CSAS, indicating that the glycosylation of the canonical site is dispensable for CSAS activity *in vitro* (Fig. 7). Finally, the predicted protein structure of *Drosophila* CSAS suggested that CSAS functions as a dimer in which the noncanonical glycosylation sites of monomers are positioned in close proximity to the dimerization region. The distance between Asn212 of one monomer and Glu169 located within another monomer’s dimerization region is estimated to be only 2.45Å (Fig. 9), which suggests that N-linked glycosylation of Asn212 can support dimerization interactions, thus promoting the stability of CSAS. The importance of the dimerization region for the biological function of human CMAS was underscored by the finding that a missense mutation in this region (R188H) causes autosomal recessive intellectual disability (8). It is also interesting to note that a missense mutation in the C-terminal domain of medaka CMAS was found to affect protein solubility and result in developmental lethality, which highlights the importance of CSAS/CMAS protein stability for *in vivo* functions (52).

Interestingly, in *Escherichia coli* expression experiments, *Aedes aegypti* CSAS with truncated signal peptide was found to have similar *in vitro* activity to the mouse ortholog (30), which contrasts with our results showing that *Drosophila* CSAS^nSP^ has undetectable *in vitro* activity. These different results may be explained by inherent stability differences between the *Aedes* and *Drosophila* proteins, or differences in expression systems, and/or assay conditions (AaCSAS was assayed in cell lysates without purification). Further investigation will need to clarify these scenarios.

Previous studies demonstrated that N-X-C sites represent suboptimal substrates for oligosaccharyltransferases and are modified at a lower frequency than canonical sites (53). Consistent with previously published data, our results indicated that a significant proportion of CSAS molecules are not glycosylated at Asn212. At the steady-state level at 25 °C, this fraction is about 40%, which likely underestimates the rate of biosynthesis of the glycoform without noncanonical site glycosylation because of its increased turnover. The inefficient glycosylation of Asn212 creates two pools of CSAS molecules within the cell which represent glycoforms with different stabilities. They are differently affected by temperature, which provides a potential mechanism for a temporal two-phase response of CSAS activity to heat shock. The initial increase in temperature is expected to quickly elevate CSAS activity within the cell because of the steep dependence of CSAS enzymatic activity on temperature (19). At the same time, more prolonged exposure to heat will result in decreasing activity because of increased turnover of the less stable pool of underglycosylated CSAS. As a poikilotherm, fruit flies need to adapt to the environmental stress associated with changes in temperature and to adjust neural transmission and behavioral responses accordingly. The initial increase of neural activity in flies caused by acute heat shock is important for escape response, while decreased neural activity in prolonged heat stress conditions is essential for energy preservation (54, 55). CSAS represents a rate-limiting step in the sialylation pathway, and thus the effect of temperature on CSAS activity is expected to translate into the effect on excitability and behavior (17, 19). It is tempting to speculate that generation of distinct protein glycoforms with different functional properties may represent a general paradigm of the function of glycosylation at noncanonical sites. Further *in vivo* studies will be required to test this hypothesis.

Our experiments shed light on the importance of subcellular localization for CSAS *in vivo* activity. We found that the CSAS^nSP^ mutant localized outside the secretory pathway compartment was not active in rescue assays (Fig. 3*B*). At the same time, the CSAS^QQ^ mutant was expressed at a similar level and similarly not glycosylated; however, it showed rescue activity in the rescue assays, which indicated that the proper localization to the secretory pathway compartment is important for CSAS function. Interestingly, when highly overexpressed, CSAS^nSP^ could partially rescue *CSAS* mutants, suggesting that it retains some enzymatic activity and that the transport of CMP-Sia into the Golgi exists in *Drosophila* cells, though it is not very efficient. Considering that CSAS is normally localized to the Golgi, *Drosophila* cells do not need a CMP-Sia transporter to deliver CMP-Sia into the Golgi for sialylation. On the other hand, CMP-Sia transporters are essential for the mammalian sialylation pathway because CMP-Sia is synthesized in mammalian cells outside of the Golgi, in the nucleus. Thus, mammalian sialylation depends on the activity of the CMP-Sia transporter, SLC35A1, that delivers CMP-Sia into the Golgi for sialyltransferase-mediated modification of different substrates (38, 56). Despite these differences in the subcellular arrangement of the sialylation pathway, the function and structure of CSAS enzymes are significantly conserved between *Drosophila* and vertebrates (13, 16, 19). Here, we demonstrated this functional conservation by *in vivo* assays using transgenic expression of the human orthologue of *Drosophila* CSAS, which revealed that human CMAS can fully rescue the paralysis phenotype of *CSAS* mutants while being localized to the nucleus (Fig. 5*B*). Once again, this result pointed at the presence of a CMP-Sia transporter activity in *Drosophila* cells. However, the molecular nature of this activity, possibly reflecting a promiscuous specificity of a yet uncharacterized nucleotide sugar transporter, remains elusive (10, 57).

## Experimental procedures

### *Drosophila* strains and cultures

Genetic strains with *CSAS* mutant alleles were previously described (16, 17, 19). Transgenic strains with Gal4 drivers and *UAS-Gal80ts* construct used in this study were obtained from the Bloomington *Drosophila* Stock Center (Indiana University, IN). Transgenic strains with *UAS-CSAS*^*WT*^*, UAS-CSAS*^*QN*^*, UAS-CSAS*^*NQ*^*, UAS-CSAS*^*QQ*^*, UAS-CSAS*^*nSP*^, and *UAS-hCMAS* were generated using the site-specific integrase PhiC31 and inserted into the same attP2 landing site on the third chromosome (23, 24). The transgenic strains were multiply outcrossed (>7X) to *w- Canton S* strain which was used as a “wild type” control. Unless indicated otherwise, all strains were reared in an incubator with controlled environment (25 °C, 60% humidity, 12-h day/night light cycles).

### Generation of CSAS mutant constructs

The *CSAS cDNA* construct tagged with 3xFLAG-encoding sequence (16) was modified using PCR-based site directed mutagenesis to obtain *CSAS* constructs with desired mutations (see Supplementary Material for details). A plasmid encoding GFP-tagged human CSAS construct was a gift from Michael Betenbaugh (The Johns Hopkins University, Baltimore, MD, USA). It included the *HsCMAS* cDNA fused in-frame with GFP-coding sequence, which results in expression of full-length human CMAS tagged with GFP at the C terminus, as previously described (13). The WT and mutagenized *CSAS* constructs and the *HsCMAS* construct were inserted into *pUASTattB* vector for *in vivo* expression using UAS-Gal4 system (22, 23).

### Transgenic expression of CSAS constructs using the TARGET system

To regulate the level of transgenic expression of CSAS constructs, we generated the genotype combining a *CSAS* null allele (*CSAS*^*221*^ (16)) with *Gli-Gal4* driver and *UAS-Gal80ts*, a construct expressing a temperature sensitive form of the Gal80 transcriptional repressor (the TARGET system (29)). These flies were crossed to flies with a genotype carrying *CSAS*^*221*^ recombined with a transgene of interest, *UAS-CSAS*^*XX*^ (where *XX* was *WT, NQ, QN, QQ,* or *nSP*). The cross and the progeny were kept at 28 °C to maintain the partial suppression of *Gli-Gal4*-mediated expression of CSAS constructs by Gal80ts. The progeny of the cross with the genotype *w∗; Gli-Gal4/+; UAS-Gal80ts CSAS*^*221*^*/UAS-CSAS*^*XX*^ *CSAS*^*221*^ were analyzed by Western blots and TS paralysis assays as described below.

### Brain dissections, immunostaining, and subcellular localization analyses

The dissection and immunostaining of larval brains were carried out essentially as previously described (58). Briefly, third instar larvae were dissected in ice-cold Ringer’s solution, and fixed in fresh fixative solution (4% paraformaldehyde, 50 mM NaCl, 0.1 M Pipes, pH 7.2) for 20 min at room temperature with gentle agitation. After extensive washes in PBT buffer (137 mM NaCl, 2.7 mM KCl, 10 mM Na_2_HPO4, 1.8 mM KH_2_PO_4_, and 0.1% Triton X-100) and blocking in PBTB (0.1% PBT and 1% bovine serum albumin) with 10% normal goat serum, the fixed brains were incubated in with primary antibodies in PBTB at following dilutions: mouse anti-FLAG M2 (Sigma-Aldrich), 1/2000; rabbit anti-GM130 (Invitrogen), 1/500; mouse monoclonal anti-Lamin C C28.26 (the Developmental Studies Hybridoma Bank, Iowa City, IA, deposited by Fisher, P.A.). This was followed by additional washes in PBTB and incubation in dark with secondary antibodies, goat anti-mouse-Cy3 (Invitrogen) and goat anti-rabbit-Alexa Fluor 488 (Invitrogen), each used at 1/250 dilution. The specificity of antibodies was validated by immunostaining experiments using negative (cells without epitope expression) and positive (cells expressing epitopes), and/or by Western blot analyses using similar control samples. Information on the validation of antibody specificities was also provided by manufacturers. After additional washes, stained brains were mounted on slides in Vectashield (Vector Laboratories). Imaging was performed by Zeiss AxioImager microscope equipped with ApoTome2 module for optical sectioning and by Leica SP8 confocal microscope using 40x and 63x objectives. Subcellular localization of CSAS variants was analyzed by FIJI (ImageJ) software essentially as described earlier (16).

### Temperature-sensitive paralysis assays

The assays were performed essentially as described previously (15, 16). Briefly, flies were collected on the day of eclosure and aged for 5 days, during which they were transferred once on day 3 to vials with fresh food. For TS assays, individual flies were transferred to empty vials, and the vials were submerged in a 38 °C temperature-controlled water bath. The paralysis was defined as a condition when a fly is down and unable to stand and walk for at least 1 min. About 20 flies were assayed for each genotype.

### Preparation of fly lysates, purification of CSAS constructs

Young adult flies (0–2 day-old) expressing FLAG-tagged CSAS constructs were homogenized in lysis buffer (50 mM Tris–HCl pH 7.6, 200 mM NaCl, 0.5% Triton X-100, 1 mM PMSF and Complete Protease Inhibitor cocktail (Roche), 40 μl per fly) on ice using Dounce homogenizer. After homogenization, crude lysates were sonicated 4 times for 5 s with 10 s intervals using Branson Sonifier 150 on ice and incubated at +4 °C with nutation for 25 min for complete lysis. Nonsoluble material was removed by centrifugation for 20 min at 18,000*g* at +4 °C. Supernatant was considered a soluble protein fraction and used for downstream applications, including protein purification using anti-FLAG agarose beads (Sigma-Aldrich), Western blot analyses, and size-exclusion chromatography. Protein concentration in lysates was determined using Pierce BCA Protein Assay Kit (Thermo Fisher Scientific, #23225) and bovine serum albumin as a standard.

For CSAS purification, 20 μl of anti-FLAG beads were washed with lysis buffer and added to 1 ml of fly lysate. After 4 to 8 h of incubation at +4 °C with nutation, beads were washed with lysis buffer and kept in storage buffer (50 mM Tris–HCl, pH 7.5, 50 mM NaCl, 0.1% Triton X-100) until used in assays.

### CSAS activity assays

CSAS activity assays were performed essentially as described previously (19). Briefly, the assays were carried out in reaction buffer (100 mM Tris–HCl pH 8.0, 20 mM MgCl_2_, 0.2 mM DTT) containing 5 mM CTP and 3 mM N-acetylneuraminic acid (Neu5Ac). To start the reactions, 100 μl of 2x reaction buffer were mixed with 100 μl of bead suspension with bound CSAS. Mixtures were centrifuged at 600*g* for 30 s, and 100 μl of supernatant was withdrawn and kept at −20 °C as a negative control. The rest of the reaction mixture with beads was incubated in 200 μl PCR tubes for 1 h at 37 °C with rotation while submerged in water bath. To stop the reaction, tubes were placed on ice for 5 min. Beads were separated from reaction solution by centrifugation at 14,000*g* for 45 min at +4 °C, and the solution was cleared by passing through 10K Amicon Ultraspin filtration units (Millipore). To quantify the amount of synthesized CMP-Neu5Ac, filtrates were analyzed by high performance anion-exchange chromatography using UltiMate 3000 HPLC system equipped with Variable Wavelength UV detector as previously described (19). Briefly, 25 μl of reaction mixture was mixed with 25 μl of 5 mM NaOH (eluent A) and injected into 4 mm × 250 mm CarboPac PA-1 column (Dionex) equilibrated with the same solution. Elution was performed by linear gradient of 1 M sodium acetate in 5 mM NaOH (eluent B) as follows: 10%, 8 min; linear gradient 10 to 50%, 16 min, linear gradient 50 to 100%, 6 min; 100%, 12 min). Elution was monitored by UV detector at 271 nm. Quantification was performed using Chromeleon 6.8 software. Commercial Neu5Ac (Sigma-Aldrich) was used as a quantification standard.

Relative activities of CSAS variants were calculated by normalizing the amount of synthesized CMP-Neu5Ac to the quantity of purified CSAS protein used in each assay, expressed in relative units based on Western blot quantification. For protein quantification, equal volumes of bead suspension containing purified protein were loaded onto gels for all samples within each biological replicate. ImageJ software was used for densitometric quantification of the Western blot signals. The relative activity of each CSAS variant was calculated by dividing the amount of CMP-Neu5Ac synthesized by the relative amount of CSAS variant in the assay (as determined above), with all values normalized to the activity of CSAS^WT^.

### Glycosidase treatment

PNGase F and Endo H enzymes were purchased from New England Biolabs (MA). Precleared lysates from young flies were used for PNGase F treatments. For Endo H treatment, we used affinity purified proteins. Preparation of lysates and CSAS purification were performed as described above. Treatment with glycosidases was carried out according to manufacturer’s protocols. After the treatment, the reaction mixtures were analyzed by Western blots.

### Western blot analysis

Proteins (15–20 μg/lane) were separated using 12% SDS-PAGE and transferred onto nitrocellulose membrane (Bio-Rad). The membrane was blocked in 5% nonfat dry milk in 1x TBST (20 mM Tris–HCl, 150 mM NaCl, 0.1% Tween 20, pH 8.0) and developed using mouse anti-FLAG antibodies (Sigma-Aldrich) 1/8000, followed by goat anti-mouse horseradish peroxidase-conjugated secondary antibodies (Jackson ImmunoResearch Labs) at dilution 1/10,000 in blocking buffer. SuperSignal Pico PLUS Chemiluminescent substrate (Thermo Fisher Scientific) and I600 imager (GE/Amersham) were used to visualize and record chemiluminescence signal. Quantification was performed using ImageJ software. For loading control, prior Western blot development, membrane was stained for total amount of loaded protein with Ponceau S, imaged, and destained with 3 × 10 min washes in 1xTBST, pH 8.0. Coomassie G250 staining of protein gels was also used in some experiments to estimate the total protein amount in lysates.

### Size-exclusion chromatography

Size-exclusion chromatography was performed using UltiMate 3000 HPLC system equipped with LPG-3400SD Standard Quaternary Pump, VWD-3100 Variable Wavelength Detector and fraction collector AFC-3000 under control of the Chromeleon 6.8 software. One hundred microliters of clarified lysate was injected into Superdex 75 10/30 HR column (GE HealthCare) using manual injector Rheodyne 7725i and eluted with 150 mM NaCl in 20 mM potassium phosphate buffer, pH 6.8 at flow rate 1 ml/min. Elution was monitored at wavelength 280 nm. Collected fractions, 0.333 ml each, were analyzed by Western blots.

### Sequence alignment and signal peptide prediction of CSAS

*Drosophila* CSAS sequences were obtained from OrthoDB database and aligned using the Clustal Omega multiple sequence alignment program (59, 60). Signal peptide prediction was carried out using SignalP-6 (61).

### 3D protein structure prediction and substrate molecular docking of CSAS

The protein three-dimensional structure of CSAS dimer was produced using a singularity container of AlphaFold2 v2.1.2 (https://github.com/google-deepmind/alphafold/releases) (34, 62). In brief, AlphaFold2 was run with the multimer model, on a single A100 (48-core) GPU, with a full database search. Two copies of the CSAS amino acid sequence in the FASTA format were included as input for AlphaFold2. A single monomeric unit of the predicted dimer structure was targeted for docking. The metal-cofactor Mg^2+^ was selected for docking based on previous work (19) with the metal ion-binding site prediction and modeling server (63). The “dock box” was determined based on the predicted CSAS dimer overlayed with CMP-N-acetylneuraminate synthetase (Protein Data Bank (PDB): 6IFD) from *V cholerae* (35) as the reference for mapping in ChimeraX (64). This mapping was used to manually define the “dock box” boundaries and depth (Table S2) with AutoDock Tools (65). Substrate structure files were prepared by downloading corresponding Structure Data Files from the PubChem database (66) for CTP (CID 6176) and Neu5Ac (CID 439197). The Structure Data Files were prepared for docking by sanitizing, adding hydrogens, and converting the files into PDBQT format. Docking was performed with AutoDock-Vina (67, 68). Briefly, Neu5Ac was docked to the CSAS predicted dimer bound with the Mg^2+^ ion, followed by CTP. Default options were used for AutoDock-Vina, except the “–exhaustiveness” flag was set to 32 (default = 8) (Table S2). The top bound conformation for each substrate was used for the final model. The fully docked structure was converted into PDB format using ChimeraX. CHARMM was used to add the M5N2 oligomannose glycans at amino acid residues N66 and N212 in both monomeric units (37). Following previously established methods (69), the highest ranked predicted model from AlphaFold2 had the multiple sequence alignment (Fig. S5), pLDDT (Fig. S6), and pAE (Fig. S7) metrics investigated to identify any areas of low model confidence. The first 21 amino acids from the N terminus in the predicted structure were truncated as their corresponding predicted local distance difference test (pLDDT) and predicted aligned error (pAE) were low and high, respectively, (Figs. S6, S7) suggesting low model confidence during the prediction refinement. The exclusion of this region from the structure is consistent with a SignalP-6 prediction which indicated that the first 21 amino acids are likely cleaved (probability ∼ 0.8) after protein translocation into the endoplasmic reticulum (61). Visualization and analysis of CSAS molecular models were carried out using the ChimeraX program (64).

### Statistical analysis

All experiments were performed using at least three biological replicates, unless indicated otherwise. Whenever possible, each experiment included at least three technical repeats. All individual data points shown in figures represent independent biological replicates. Statistical analyses in experiments with multiple groups of data were performed by one-way ANOVA with Tukey *post hoc* comparisons. In all figures, ∗, ∗∗, ∗∗∗, and ∗∗∗∗ indicate *p* values of <0.05, <0.01, <0.001, and 0.0001, respectively; NS indicates no significant differences found (*p* > 0.05). Unpaired two-tailed *t* test was used for experiments with two groups of data. The sample size required for reliable statistical analyses was determined empirically, based on previous experience and knowledge of the system. GraphPad Prism software was used for statistical analyses.

## Data availability

Data described in the manuscript are shown in the figures and included in Supplementary Material. Detailed protocols and the transgenic *Drosophila* strains generated in the study are available per request.

## Supporting information

This article contains supporting information (60).

## Conflict of interest

The authors declare that they have no conflicts of interest with the contents of this article.
