# Supplementary material for: CMP-sialic acid synthetase in Drosophila requires N-glycosylation of a noncanonical site
Source: J Biol Chem. 2025 Apr 7;301(6):108483. doi: 10.1016/j.jbc.2025.108483 (PMC12144448; doi:10.1016/j.jbc.2025.108483)
Supplement: Supporting information [file mmc1.pdf]

CMP-sialic acid synthetase in *Drosophila* requires N-glycosylation of a non-canonical site.

Boris Novikov, Devon J. Boland, Ilya Mertsalov, Hilary Scott, Saniya Dauletbayeva, Pedro Monagas-Valentin, and Vladislav Panin.

Supplementary Information

Supplementary Experimental Procedures

Genotypes of Drosophila strains used in the study.

WT, w* Canton S - control genotype and the genetic background used in all experiments (“wild-type”)
CSAS constructs expressed in wild-type genetic background:
w*; Act5C-Gal4/+; UAS-CSAS^{XX}/+ (where XX is WT, NQ, QN, QQ, or nSP)
CSAS constructs expressed in CSAS mutants:
CSAS^{-} Act5C>_ , w*; Act5C-Gal4/+; CSAS^{221}/CSAS^{221}
CSAS^{-} Act5C > CSAS^{XX}, w*; Act5C-Gal4/+; UAS-CSAS^{XX} CSAS^{221}/CSAS^{221} (where XX is WT, NQ, QN, QQ, or nSP)
CSAS^{-} Gli >_, w*; Gli-Gal4/+; UAS-Gal80ts CSAS^{221}/CSAS^{221}
CSAS^{-} Gli > CSAS^{XX}, w*; Gli-Gal4/+; UAS-Gal80ts CSAS^{221}/ UAS-CSAS^{XX} CSAS^{221} (where XX is WT, NQ, QN, QQ, or nSP)

Supplementary Figures and Tables

Figure S1. Sequence alignment between Drosophila CSAS and human CMAS. Asterisks indicate N-glycosylation sites found in CSAS. They are not conserved in the human counterpart. Arrowhead indicates the predicted cleavage site of CSAS signal peptide, also showing the site of N-terminal truncation in $CSAS^{nSP}$. The C-terminal domain present in human CMAS is absent from Drosophila CSAS and not shown in the alignment. The alignment was created by Clustal Omega server at EMBL (1).

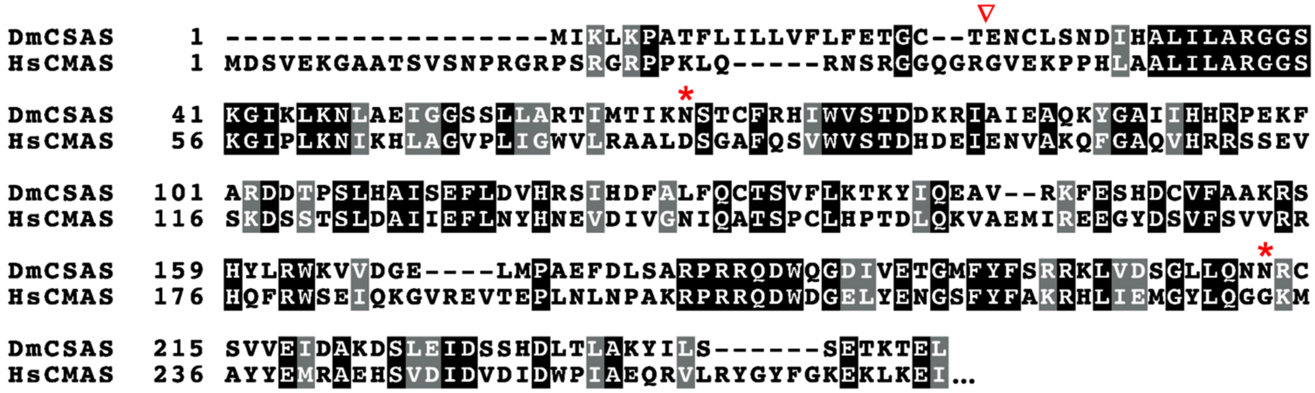

Figure S2. Endoglycosidase H treatment of purified WT and nSP variants. The treatments of $CSAS^{WT}$ results in removal of both N-glycans, which is evident from the collapse of the two bands representing different CSAS glycoforms into one band with a smaller molecular mass of approximately the same size as $CSAS^{nSP}$. The nSP variant is not glycosylated due to its localization outside of the secretory compartment, which is consistent with the absence of Endo H treatment effect on this variant. C_H and C_L indicate heavy and light IgG chains, respectively, that leached from FLAG affinity beads during CSAS elution.

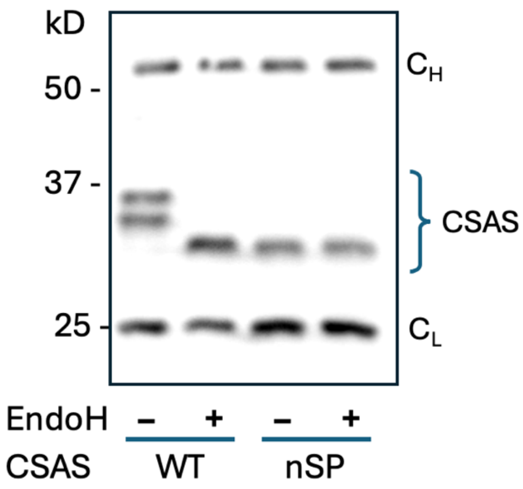

Figure S3. Western blot analysis of size exclusion chromatography fractions of cell lysates from flies with transgenic expression of CSAS-FLAG. The fractions were analyzed by SDS-PAGE using a 4-20% gradient Tris-Glycine gel (Thermo Fisher).

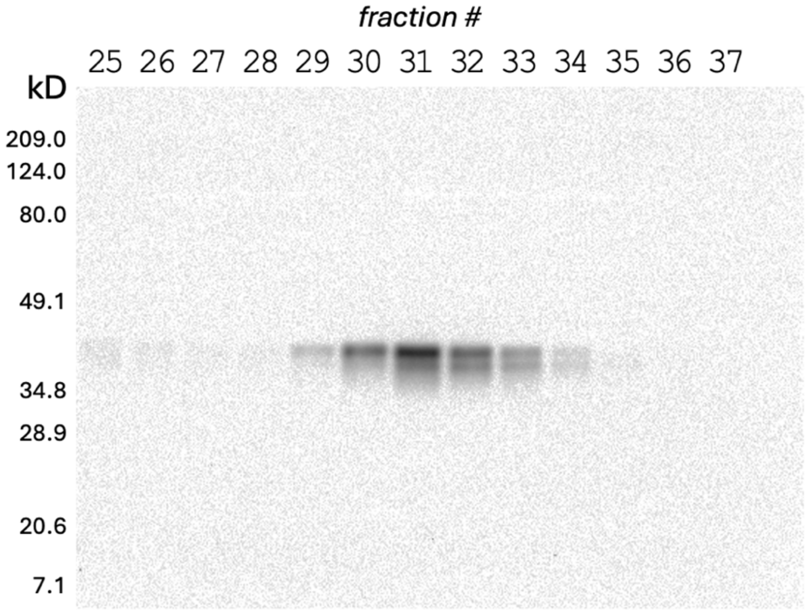

Figure S4. Evolutionary conservation of noncanonical N-glycosylation site among different arthropods. The conserved non-canonical site is highlighted. The alignment was generated using the NCBI COBALT online tool. Sequence accession numbers: NP_730474.2 (*Drosophila melanogaster),* XP_001663017.1 *(Aedes aegypti),* XP_053673996.1 *(Anopheles nili),* XP_063361988.1 *(Cydia amplana),* XP_050686206.1 *(Eriocheir sinensis),* XP_037823820.1 *(Lucilia sericata),* XP_055676721.1 *(Lutzomyia longipalpis),* XP_045448656.1 *(Papilio machaon),* XP_013143704.1 *(Papilio polytes),* XP_059613060.1 *(Phlebotomus argentipes),* XP_055713317.1 *(Phlebotomus papatasi),* XP_063842754.1 *(Scylla paramamosain),* XP_047529755.1 *(Vanessa Atalanta).*

Drosophila melanogaster (fruit fly)	203	LVDSGLLQN**N**RCSVVEIDAKDSL	225...
Aedes aegypti (yellow fever mosquito)	219	LLLEGRFQN**N**NCEVVVIDERDSL	241...
Anopheles nili (Theobald mosquito)	215	IIEANSFQN**N**RCSVVEVEEIDAL	237...
Phlebotomus argentipes (sand fly)	211	LLDRGVFQT**N**SCGFVEIPSQDAL	233...
Phlebotomus papatasi (sand fly)	217	LIKRGVFQK**N**SCKFVEIEPEDSL	239...
Lutzomyia longipalpis (sand fly)	215	LLDLGIFQT**N**SCKFVEIDPEDSL	237...
Lucilia sericata (blowfly)	201	LVSKNSLQD**N**RCAIVEVDAQDAI	223...
Cydia amplana (rusty oak moth)	197	VVMSGSYQN**N**NCSVWEVSAAESF	219...
Melitaea cinxia (Glanville butterfly)	195	VIVKGTLQN**N**NCTVWELSQAESL	217...
Papilio machaon (swallowtail butterfly)	199	LIKQGLLQN**N**NCTVFETKPKESL	221...
Papilio polytes (mormon butterfly)	194	LIKQGILQN**N**NCTVLETNAKESL	216...
Vanessa Atalanta (red admiral butterfly)	195	LIIKGYLQN**N**NCTVWEISQEESL	217...
Eriocheir sinensis (Chinese mitten crab)	187	LVKSGLLQG**N**RCGYVEVPQEDSL	209...
Scylla paramamosain (mud crab)	187	VLQKGLLQG**N**RCGYIEVPQEDSL	209...

Figure S5. 2D heat-map, scatter plot of the total multiple sequence alignment of the CSAS dimer fasta sequence from the databases AlphaFold2 searched against in the initial MSA generation step. Colored segments of heatmap represent the level of sequence identity to the target sequence (y-axis) to the query sequence (x-axis). Color scale indicates level of identity, while white (empty) indicates no alignment. The black trace through the heatmap indicates the density of aligned sequences to a given position of the query sequence.

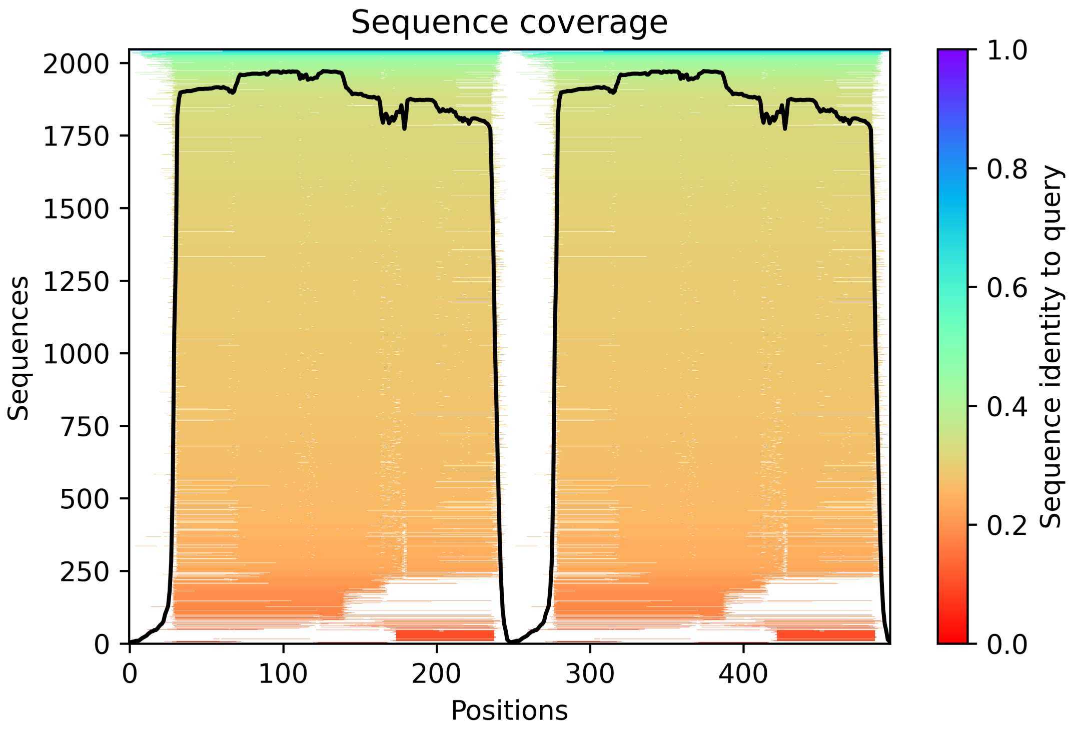

Figure S6. 2D heat-map, scatter plot of the predicted local distance difference test (pLDDT) values for each residue in the predicted 3-dimensional structure of CSAS. Individual data points are colored by the respective pLDDT values, with red being low pLDDT (<25) to blue being high (>75).

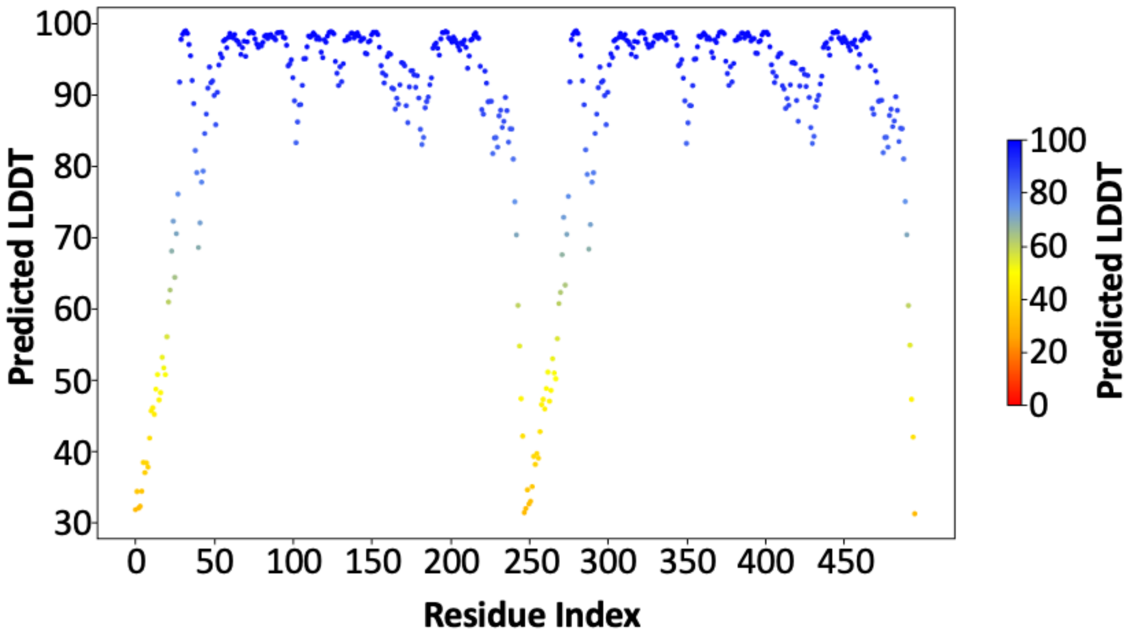

Figure S7. 2D heat map of the predicted aligned error in Å for each pair-wise residue combination within the predicted 3-D structure of CSAS. Each monomeric unit of the predicted dimeric structure is visualized by each box where the line y=x passes through. Bins are colored by their pAE values from blue (low) to yellow (high).

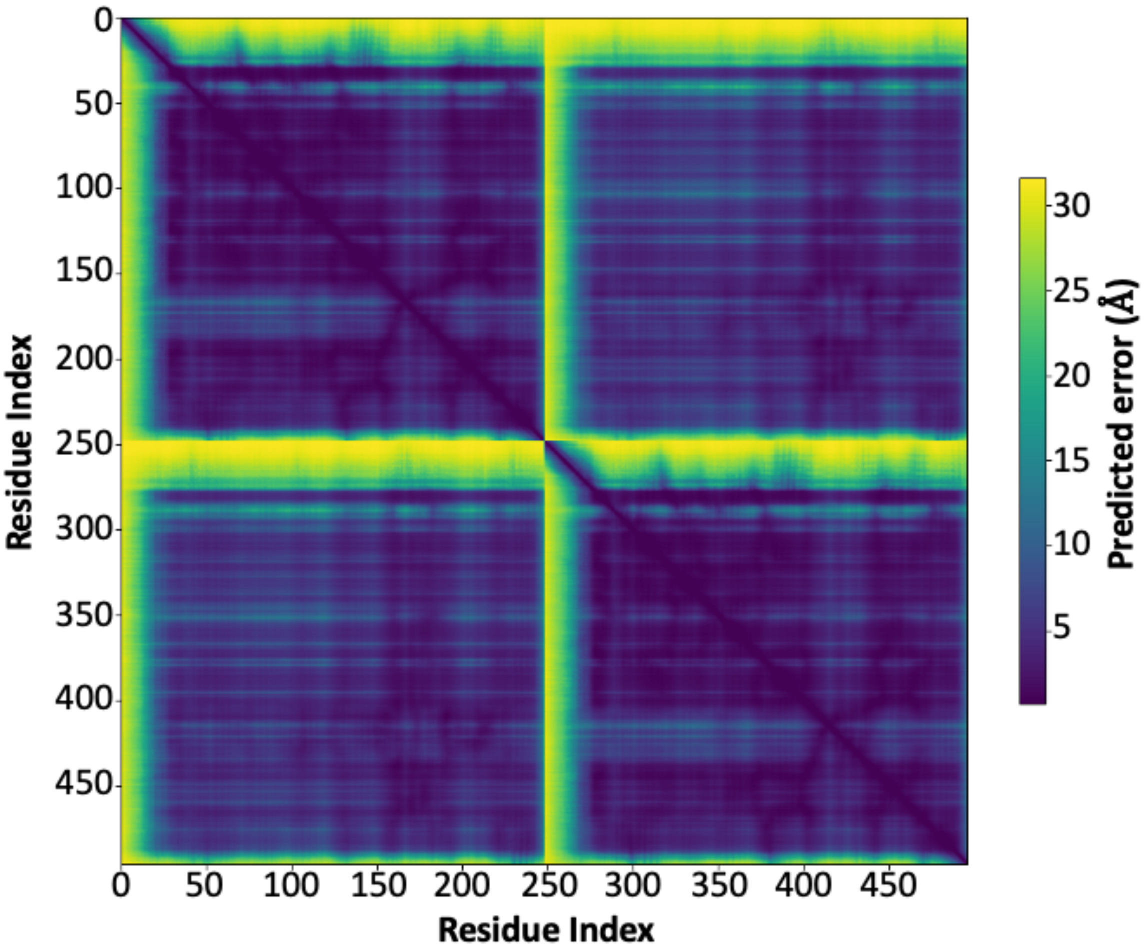

Table S1. PCR primers used for site-directed mutagenesis of CSAS.
Mutagenized bases are not capitalized.

Primer name	*Sequence*	*Amino acid changes*
CSAS-Q66-FRW	5'-TGACCATCAAAAcAgTCGACTTGCTTTCGACAT-3'	N66Q
CSAS-Q66-REV	5'-AAGCAAGTCGAcTgTTTGATGGTCATAATTGTG-3'	N66Q
CSAS-Q212-FRW	5'-TACTGCAAAACcAgCGCTGCTCCGTTGTGGAAA-3'	N212Q
CSAS-Q212-REV	5'-ACGGAGCAGCGcTgGTTTTGCAGTAGCCCAGTG-3	N212Q
CSAS-nSP-FRW	5'-GAAGGTACCAAAAtGGAAAATTGTCTGAGCAACGATATTCATGC-3'	Δ1-21,T22M
CSAS-nSP-REV	5'-TCCTCTAGATTTACTTGTCATCGTCATCC-3'	-

Table S2. Formatted output from docking CTP and Neu5Ac with AutoDock-Vina. Each substrate's top 6 modes (conformers) and the corresponding simulated affinities are represented in kcal/mol. The fourth column includes the dimensions of the "Dock Box" used in the docking process, and are expressed in Å.

**Substrate**	**CTP**	**Neu5Ac**	**"Dock Box" Boundaries**
**Conformer 1**	-7.19	-6.73	center_x = -4.972
**Conformer 2**	-7.029	-6.655	center_y = 20.861
**Conformer 3**	-6.903	-6.557	center_z = 0.000
**Conformer 4**	-6.884	-6.538	size_x = 68
**Conformer 5**	-6.843	-6.471	size_y = 64
**Conformer 6**	-6.713	-6.458	size_z = 56

Supplementary References

1. Madeira, F., Madhusoodanan, N., Lee, J., Eusebi, A., Niewielska, A., Tivey, A. R. N. *et al.* (2024) The EMBL-EBI Job Dispatcher sequence analysis tools framework in 2024 Nucleic Acids Res **52**, W521-W525
